# Altered intestinal microbiota in patients with chronic pancreatitis: implications in diabetes and metabolic abnormalities

**DOI:** 10.1038/srep43640

**Published:** 2017-03-03

**Authors:** Sai Manasa Jandhyala, A. Madhulika, G. Deepika, G. Venkat Rao, D. Nageshwar Reddy, Chivukula Subramanyam, Mitnala Sasikala, Rupjyoti Talukdar

**Affiliations:** 1 Division of Basic Sciences, Asian Healthcare Foundation, Hyderabad, India; 2 Dept. of Clinical Nutrition, Asian Institute of Gastroenterology, Hyderabad, India; 3 Dept. of Biochemistry, Asian Institute of Gastroenterology, Hyderabad, India; 4 Dept. of Surgical Gastroenterology, Asian Institute of Gastroenterology, Hyderabad, India; 5 Dept. of Medical Gastroenterology, Asian Institute of Gastroenterology, Hyderabad, India.

## Abstract

Intestinal dysbiosis and its functional implications in chronic pancreatitis (CP) have not been elaborately studied. We evaluated the taxonomic and functional alterations in intestinal microbiota in 30 well-characterised patients with CP (16 without, 14 with diabetes) and 10 healthy controls. The patients with CP and diabetes had significantly longer disease duration and greater degree of malnutrition. There was increase in plasma endotoxin concentrations from controls to CP non-diabetics to CP diabetics. We observed significant differences in richness and alpha diversity between the groups. We also observed increase in the Firmicutes:Bacteroidetes ratio in CP patients without and with diabetes. There was reduction in abundance of *Faecalibacterium prausnitzii* and *Ruminococcus bromii* from controls to CP non-diabetics to CP diabetics. On the other hand, there was increase in LPS (endotoxin) synthetic pathways (KEGG orthology) in the groups. *Faecalibacterium prausnitzii* abundance correlated negatively with plasma endotoxin and glycemic status; while plasma endotoxin correlated positively with blood glucose and negatively with plasma insulin. Our results have important implications for future studies exploring mechanistic insights on secondary diabetes in CP.

Chronic pancreatitis (CP) is characterised by abdominal pain, reduction in digestive enzyme secretion (pancreatic exocrine insufficiency) and endocrine dysfunction/diabetes (DM)^1^. Pancreatic exocrine insufficiency (PEI) results in maldigestion of fat and other nutrients thereby culminating in malnutrition and metabolic abnormalities. DM secondary to CP is distinct from Type I and Type II DM; and is characterised by insulin deficiency coupled with absence of glucagon and pancreatic polypeptide regulatory responses, and hepatic insulin resistance^2^,^3^,^4^. Even though oral microbial dysbiosis and small intestinal bacterial overgrowth (SIBO) has been reported in patients with CP^5^,^6^, the detailed alterations in the taxa of the intestinal microbiota in this illness has not been studied. We hypothesized that nutrient maldigestion in CP could result in alteration of the gut microbiota which could eventually contribute to the related metabolic abnormalities. With this premise, we conducted the current study, wherein we evaluated for alterations in the intestinal microbiota and their associations with metabolic abnormalities, including DM, in patients with CP.

## Results

### Patient characteristics

**Figure 1:**
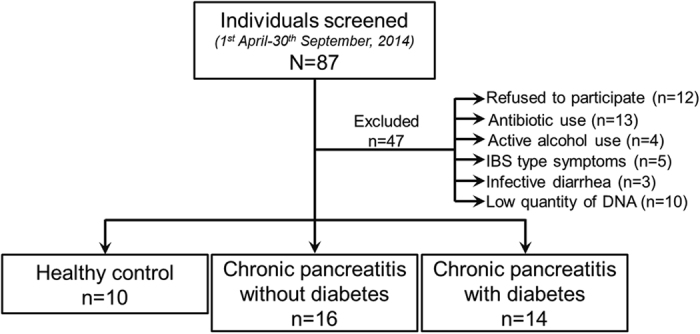
Study flow and patient distribution.

As shown in Fig. 1, we screened 87 individuals during the study period, of which 40 (10 healthy controls, 16 CP without DM, 14 CP with DM) were enrolled. Table 1 shows the demographic, morphologic, nutritional and diabetic characteristics of the individuals in the different groups. Patients having CP with DM had a significantly longer duration of illness compared to those without DM; and the CP diabetic patients had low plasma C-peptide levels [Mean (SD) 1.3 (0.5) ng/ml; Reference range: 1.1–4.4 ng/ml]. Mean (SD) age of onset of DM was 27.7 (13.9) yrs. As indicated by the subjective global assessment (SGA) scale, a significantly higher proportion of patients with DM had severe malnutrition compared to patients without DM (p = 0.003). Furthermore, CP patients with DM had significantly lower pre-albumin (p < 0.001), transferrin (p < 0.001) and vitamin D (p < 0.001) concentrations in circulation. Supplementary Table S1 shows the details of dietary intake of the individuals in the three groups.

### Bacterial metagenomic characteristics and diversity

**Figure 2:**
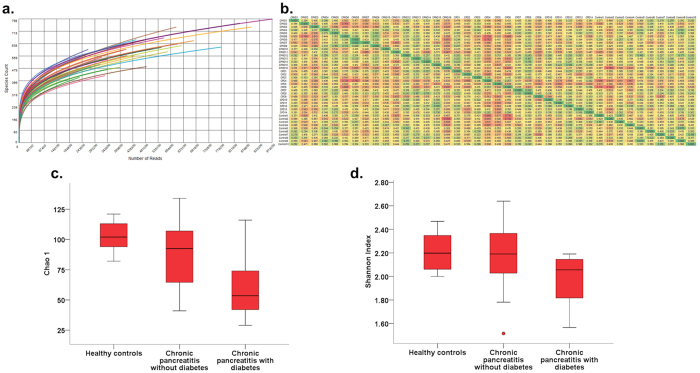
Bacterial richness and diversity alters with progression of CP. **(a)** Rarefaction curves showing of individual samples from each group; Box and Whisker plot indicating; **(b)** Heatmap indicating lower beta diversity (between diabetic chronic pancreatitis patients compared to the other groups; **(c)** reduction of bacterial richness (Chao 1) from healthy controls to chronic pancreatitis patients without to those with diabetes (Kruskal Wallis test, [p(corr.) = 0.01]) and **(d)** reduction of Shannon’s alpha diversity in patients having chronic pancreatitis with diabetes (Kruskal Wallis test, [p(corr.) = 0.04].

Supplementary Table S2 shows the post quality control sequence count and length, metagenomic quantity and quality, richness and alpha diversity for each individual. Figure 2a shows the individual rarefaction curves of controls and patients; and Fig. 2b shows the beta diversity (Whittaker’s), which was lower in patients with CP who had DM compared to the other two groups. As shown in Fig. 2c, there was reduction of the richness (Chao1 index) from control individuals to non-diabetic CP to diabetic CP patients [p(corr.) = 0.01]; while Shannon index was lower in the diabetic CP patients compared to controls and CP patients without CP [p(corr.) = 0.04] (Fig. 2d). Individual comparisons of diversity indices between healthy controls, CP with and CP without diabetes are shown in Supplementary Table S3. These observations indicated reductions in the bacterial richness and diversity with increasing disease duration and severity.

### Principal component analysis (PCA) and taxonomic differences

**Figure 3:**
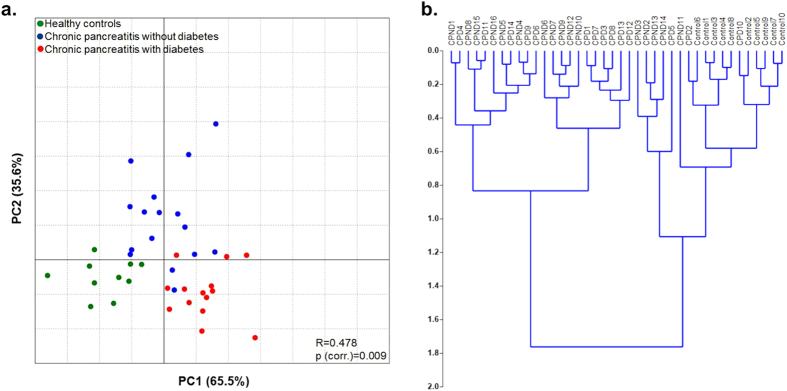
Clustering of gut microbiota at species level taxa among the study groups. **(a)** Principal co-ordinate analysis (PCA) of gut microbial species abundance showing distinct clustering in the three study groups. The relation between the microbiota and disease status was assessed with ANOSIM using Monte Carlo simulations with 10,000 replications [p(corr.) = 0.009]. **(b)** Dendrogram showing hierarchical cluster analysis based on Euclidean similarity matrix (with boot strapping to 1000) to measure closeness between individual samples.

PCA showed distinct clustering of healthy controls, CP patients without and those with diabetes at the species level taxa [R = 0.478; p(corr.) = 0.009] (Fig. 3a). This was confirmed by the hierarchical clustering of individual samples within the three groups [cophenetic correlation coefficient (rc) = 0.608)] (Fig. 3b).

**Figure 4:**
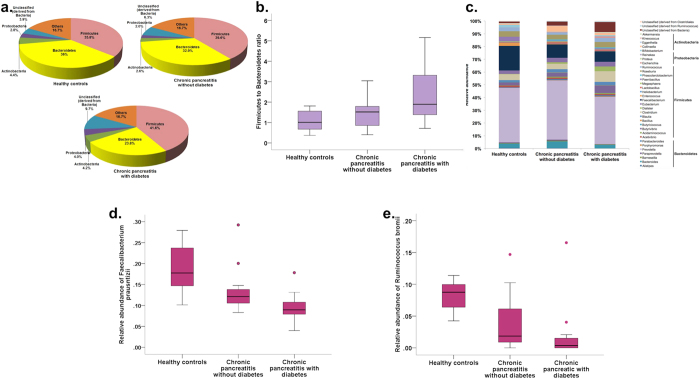
Relative abundances of gut microbiota at different taxonomic levels between the three groups. **(a)** Pie charts showing the microbial abundances in different groups at the phylum level; **(b)** Box and Whisker plot showing Firmicutes:Bacteroidetes ratio; **(c)** Stacked bars showing bacterial abundances at the genus level (genera that had a relative abundance of >1% in at least 10% of samples have been included in the figure). Box and Whisker plots showing **(d,e)** statistically significant reduction in relative abundances of *Faecalibacterium prausnitzii* (p = 0.001) and *Ruminococcus bromii* (p = 0.001) respectively.

We observed a total of 11 phyla, of which 76.8% were constituted across the study groups by Firmicutes (39.0%), Bacteroidetes (31.0%), Actinobacteria (3.8%) and Proteobacteria (2.9%). As shown in Fig. 4a,b respectively, there was a decrease in the abundance of the phylum Bacteroidetes from controls to non-diabetic CP to diabetic CP patients [p(corr.) = 0.04], and corresponding increase in the Firmicutes:Bacteroidetes (F:B) in the three groups [p(corr.) = 0.04]. There was also a trend towards statistically significant increase in unclassified organisms (derived from bacteria) in non-diabetic CP and CP with diabetes [p(corr.) = 0.06]. There were no differences in abundance in the class, family, order level taxa (Supplementary Fig. S1a–c). Even though there was reduction in Faecalibacterium at the genus level in non-diabetic and diabetic CP patients when compared to controls, this did not reach statistical significance [p(corr.) = 0.09] (Fig. 4c). However, we observed significant increase in the abundance of unclassified bacteria (p < 0.001) from healthy controls to CP without DM to CP with DM (Fig. 4c). At the species level, we observed statistically significant reduction of *Faecalibacterium prausnitzii* [p(corr) = 0.001] and *Ruminococcus bromii* [p(corr.) = 0.001] abundances from controls to non-diabetic CP to diabetic CP patients (Fig. 4d,e; Supplementary Table 3).

### Functional differences between the study groups

**Figure 5:**
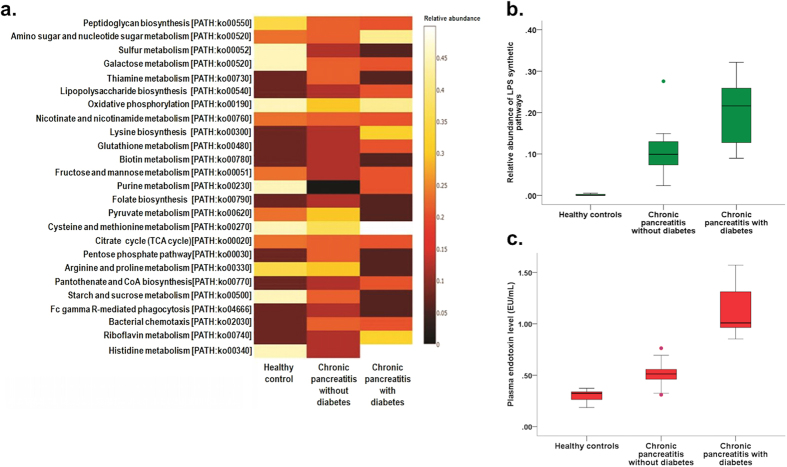
Alteration in gut microbial functions in patients with CP without and with diabetes. **(a)** Heatmap showing differences in functional pathways according to KEGG orthology (KO) between the three study groups. **(b,c)** Box and Whisker plots showing increasing relative abundances in LPS synthetic pathways **(b)** in the three groups with corresponding increase in plasma endotoxin levels **(c)**.

Next, we evaluated the functional pathways using Kyoto Enclyopaedia of genes and genomes (KEGG) orthology (KO) in the three groups and their correlations with bacterial abundance. Figure 5a shows the differences in the abundances of different metabolic pathways between the groups. We observed significant increase in lipopolysaccharide (LPS) synthetic pathways from controls to non-diabetic to diabetic CP patients [p(corr.) = 0.004] (Fig. 5b; Supplementary Table 3). There was also concomitant parallel increase in the plasma endotoxin levels (EU/ml) in the corresponding groups [p(corr.) < 0.0001] (Fig. 5c; Supplementary Table 3).

### Correlation of intestinal dysbiosis with host metabolic functions

**Figure 6:**
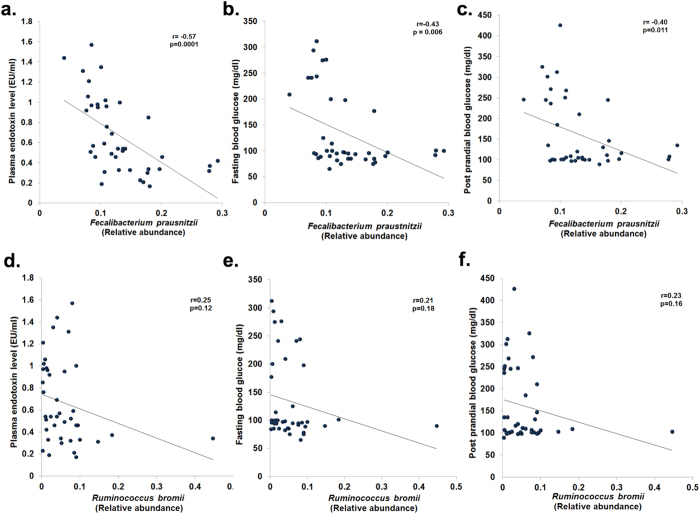
Changes in the relative abundances of bacterial species and correlations with host biochemical alterations. Scatter plots showing significant negative correlation (Pearson’s) between abundance of *Faecalibacterium prausnitzii* and **(a)** plasma endotoxin levels **(b)** fasting blood glucose, and **(c)** post prandial blood glucose levels; and between abundance of *Ruminococcus bromii* and **(d)** plasma endotoxin levels, **(e)** fasting blood glucose, and **(f)** post prandial blood glucose levels.

**Figure 7:**
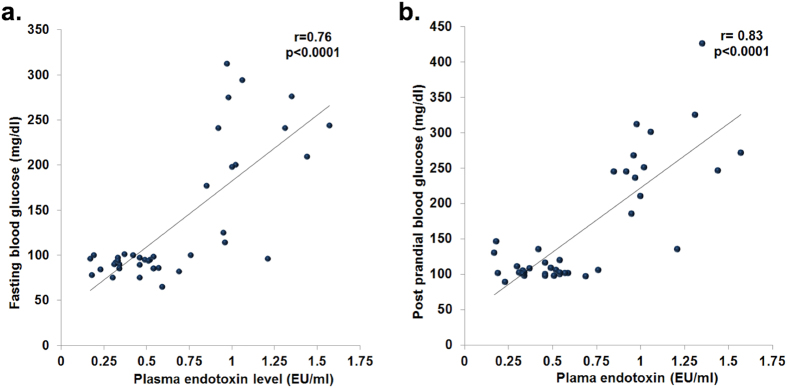
Plasma endotoxin levels correlate with host blood glucose. Scatter plots showing statistically significant positive correlation of plasma endotoxin with **(a)** fasting blood glucose and **(b)** post prandial blood glucose.

We further evaluated the correlations of bacterial genera with metabolic parameters in controls and patients. We observed significant negative correlation between relative abundance of *Faecalibacterium prausnitzii* with plasma endotoxin levels (r = −0.57; p = 0.0001), fasting blood glucose (r = −0.43; p = 0.006) and post-prandial blood glucose (r = −0.40; p = 0.011) (Fig. 6a–c). There was also a significant positive correlation of *Faecalibacterium prausnitzii* abundance with plasma insulin levels (Supplementary Fig. S2). However, there was no correlation of *Ruminococcus bromii* abundance with plasma endotoxin and blood glucose (Fig. 6d–f). On the other hand, we observed significant positive correlation of plasma endotoxin with fasting and post-prandial blood glucose levels (r = 0.76; p < 0.0001 and r = 0.83; p < 0.0001 respectively) (Fig. 7a,b).

**Figure 8:**
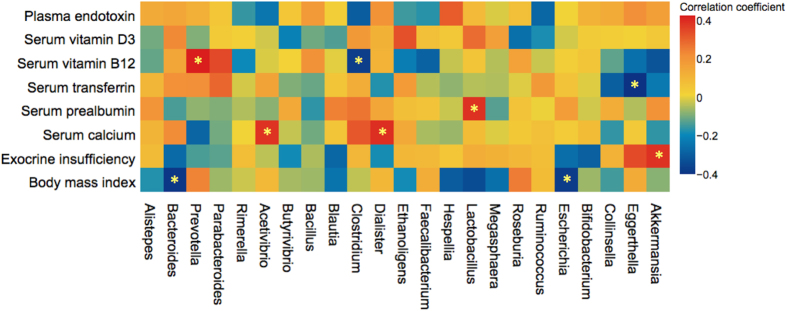
Relative abundances of genus level taxa correlates with host nutritional and metabolic functions. Heatmap showing the correlations between bacterial genera with nutritional characteristics and pancreatic exocrine insufficiency.

Other clinically relevant correlations were (Fig. 8): negative correlation of Bacteroides and Escherichia with body mass index (BMI) [(r = −0.39; p = 0.033) and (r = −0.38; p = 0.037) respectively]; negative correlation of Akkermansia with PEI (r = −0.46; p = 0.011); positive correlation of Lactobacillus with serum pre-albumin (r = 0.38; p = 0.044); positive correlation of Prevotella with serum vitamin B12 (r = 0.51; p = 0.005); negative correlation of Clostridium with serum vitamin B12 (r = −0.38; 0.041); and positive correlation of Shigella with plasma endotoxin level (r = 0.53; p = 0.003).

## Discussion

In this study we have shown significant association of intestinal dysbiosis with host metabolic functions, including diabetes, in patients with CP.

The CP patients with DM had a significantly longer disease duration and higher degree of malnutrition compared to the CP patients without diabetes. This indicates that the changes in the intestinal microbiota in our patients were dependent on disease duration and severity. We included healthy family members of enrolled patients with CP as controls so that the dietary, environmental, and genetic confounders^7^,^8^,^9^,^10^ could be controlled. Absence of any alteration of pancreatic morphology and other metabolic abnormalities in the controls was further confirmed by transabdominal ultrasonography and relevant biochemical tests.

One of our key findings was a reduction in the abundance of *Faecalibacterium prausnitzii* from healthy controls to non-diabetic CP to CP with diabetes. There was also a negative correlation between the abundance of this organism with circulating endotoxin levels. We further observed an increase in the LPS (endotoxin) synthetic pathways in the patients without and with DM. *Faecalibacterium prausnitzii* is among the most abundant commensals in the human intestine^11^. It is an acetate using butyrate producer that facilitates nutrients to the colonic epithelial cells thereby promoting their proliferation and growth^12^. It also exerts anti-inflammatory properties by inducing IL-10 and regulating T-cell responses in the intestine^13^. Studies have also shown that *Faecalibacterium prausnitzii* could improve gut barrier function by stimulating the synthesis of mucin and tight-junction proteins^12^,^14^,^15^. Therefore, reduction in the abundance of *Faecalibacterium prausnitzii* in our patients with CP was likely to have impaired the intestinal mucosal barrier integrity in a duration dependent manner.

We also observed reduction in abundance of the amylolytic organism *Ruminococcus bromii* in patients with non-diabetic and diabetic CP patients. *Ruminococcus bromii* bears a unique capability to degrade especially enzymatic digestion resistant starch, a function that is poorly seen in other starch degraders^16^. This organism has been identified as a keystone species for starch degradation in the human colon; and the butyrate and the energy harvested in the process is distributed to the colonic epithelial cells and to other bacteria^17^. Therefore, reduction of *Ruminococcus bromii* was likely to have contributed to the disruption of gut mucosal barrier and altered bacterial metabolism within the intestinal epithelial-bacterial ecological system.

Abundance of the LPS synthetic pathways and plasma endotoxin levels were higher in our patients without and with DM, despite the reduction in abundance of the phylum Bacteroidetes, which includes primarily Gram-negative organisms (source of LPS). It is likely that the majority of the unclassified phyla and genera derived from bacteria were Gram negative, resulting in increasing LPS synthetic pathways and plasma endotoxin as a function of duration and severity of the disease.

The other key finding in our study was a significant negative correlation of the abundance of *Faecalibacterium prausnitzii* with fasting and postprandial blood glucose. The validity of this result was substantiated by the significant positive correlation of the abundance of *Faecalibacterium prausnitzii* with plasma insulin levels. There was also significant positive correlation of plasma endotoxin with fasting and postprandial blood glucose. It is known that LPS could induce inflammation in beta cells via Toll like receptors (TLRs) and nuclear factor of kappa-B (NFk-B) thereby resulting in beta cell dysfunction^18^. We had earlier reported Th-cell dysregulation and cytokine mediated beta-cell dysfunction in patients with CP demonstrating a graded increase in circulating Th1 cells in CP patients without and with DM; as well as co-localization of Th1 and Th17 cells within the pancreatic islets^19^. We also demonstrated earlier an increase in IFN-γ concentration, among other cytokines, within the pancreas in patients with CP^20^. Mechanistic link between these findings and the observed results from the current study needs to be investigated and validated further.

It has been shown that diabetes^21^ and other factors such as diet, alcohol, and antibiotics could alter gut microbiota^22^. In may appear that intestinal dysbiosis in our study could have been influenced by these factors. However, the microbial taxonomic and functional abnormalities that were observed our patients with CP and diabetes were also observed in CP without diabetes, though at a lesser magnitude but significantly higher than controls. There were no differences in diet and alcohol intake between the different groups in our study. Therefore, these factors were unlikely to have skewed the gut microbiota data in our study population.

Even though our results demonstrated discrete changes in the intestinal microbiota that were associated with metabolic changes of CP, there were few limitations in the study. The sample size was small and we could not perform any formal sample size calculation since there were no previous quantitative data on the intestinal microbiota in patients with CP. However, our selection criteria were strict and we also characterised the disease phenotype in detail. We believe these steps had circumvented the sample size limitation. We also did not perform any metabolome assessment (including short chain fatty acids) in this study. However, functional analyses based on KEGG orthology using the metagenomic data provided plausible results that explain the observed association between the intestinal dysbiosis and phenotypic alterations. Furthermore, we did not perform a pancreatic polypeptide response assay, which could have conclusively confirmed that our patients had pancreatogenic diabetes. We believe these patients did have pancreatogenic diabetes since diabetes developed at a younger age (mean 27.7 yrs), with a consistent lag period after the onset of CP; and the C-peptide levels were low. Of note, CP and secondary diabetes develops earlier in India compared to that seen in the west^23^,^24^,^25^.

Treatment of CP is currently restricted to palliation of pain, supplementation of pancreatic enzymes, and control of diabetes^1^,^26^. There is still no definitive cure for this difficult to treat disease; nor are any validated strategies available to prevent disease progression that results in endocrine dysfunction, exocrine insufficiency or malnutrition^27^. Our results indicate that gut microbial manipulation strategies has the potential to emerge as a measure to prevent or delay the development of metabolic derangements including diabetes in patients with CP. Further well-designed clinical and experimental studies that would validate our data and elucidate the mechanistic insights could eventually result in translation of our results to clinical practice.

## Material and Methods

### Patient recruitment, assessment and definitions

We initiated this study after approval from the Institutional Review Board of the Asian Institute of Gastroenterology (IRB approval no. AIG/AHF IRB: 26/2013; dated 15/11/2013); and obtained written informed consent from all enrolled cases and controls. All experimental procedures were performed according to standard guidelines and procedures that were approved by the above mentioned Institutional Review Board.

We screened adult patients of both genders with CP who presented to the Pancreas Clinic at the Asian Institute of Gastroenterology, Hyderabad, India for eligibility over a 6-month period (April-September 2014). Supplementary Fig. S3 summarizes the study design with inclusion and exclusion criteria. We included patients with documented CP with at least 3 years duration, with and without diabetes. Healthy family members who were living together with the patients for at least 10 years were enrolled as controls. Exclusion criteria included: antibiotic intake in the past 3 months, history of co-morbidities (irritable bowel syndrome, inflammatory bowel diseases, chronic liver disease, obesity, and recent infective diarrhea), active alcohol use in the past 6 months; and poor quality fecal metagenomic DNA. Glucose intolerance and diabetes was diagnosed as per American Diabetic Association (ADA) 2014 criteria^28^.

Detailed clinical history from the cases and controls was recorded and disease morphology was evaluated by cross sectional imaging [contrast enhanced computed tomography (CECT) or magnetic resonance cholangiopancreatography (MRCP)] or endoscopic retrograde cholangiography (ERCP). All controls underwent transabdominal ultrasonography for evaluating the pancreas and the liver.

All subjects were subjected to routine biochemical evaluation and nutritional assessment including dietary history, anthropometric measurements, serum transferrin, serum prealbumin, serum vitamin B12, serum vitamin D, serum calcium, and haemoglobin levels. Oral glucose tolerance test (OGTT), plasma C-peptide, plasma insulin and glycosylated haemoglobin (HbA1c) were assessed to define the glycemic status of the subjects. Composite nutritional status was expressed according to the Subjective Global Assessment scale^29^. In addition, plasma endotoxin levels were also measured. Fresh stool samples were used to isolate bacterial DNA and estimate fecal elastase. Patients with steatorrhea or fecal elastase value of <200 μg/g was considered to have PEI^30^.

### Biochemical assays

OGTT was performed with a 75 g glucose load and blood glucose was measured using the Glucose Oxidase-Peroxidase (GOD-POD) method employing a commercial kit (ERBA Glucose kit; Transasia Bio-Medicals Ltd, HP, India). HbA1c was measured by high performance liquid chromatography (HPLC) using a National Glycohemoglobin Standardised Program (NGSP)^31^ certified automated analyser (Bio-Rad).

Plasma insulin and C-peptide estimation were performed using a Sandwiched Electrochemical Immunoassay (ECLIA) technique in the ROCHE Cobas‘e’ 601 Immunoassay Analyzer. Briefly, the samples were incubated initially with 20 μl of biotinylated monoclonal antibodies (anti-insulin and anti C-peptide), followed by incubation with streptavidin coated microparticles. The bound microparticles were then magnetically captured onto the surface of the electrode that resulted in induction of chemiluminescent emission which was measured on a photomultiplier and quantified by 2-point calibration with a master curve.

Serum concentrations of vitamins D and B12 were estimated by ECLIA method in the ROCHE Cobas’e’ 601instrument; serum transferrin and pre-albumin were analyzed by immunoturbidimetry on the Random Imola fully automated analyzer; and serum calcium contents were determined using the metallochromatic indicator Arsenazo III on the fully automated UniCelDxC 800 (Beckman Coulter Inc.) analyser. All assays were performed as per manufacturer’s instructions.

### Plasma endotoxin assay

Plasma endotoxin levels were estimated using the Pierce LAL Chromogenic Endotoxin Quantitation Kit (Thermo Scientific; Cat. no. 88282) as per manufacturer’s instructions^32^. Briefly, 50 μL of each standard and study samples were incubated for 5 minutes at 37 °C, followed by addition of 50 μL of Limulus Amebocyte Lysate (LAL) to each well and further incubation for 10 minutes. Subsequently, 100 μL of substrate solution was added to each well, and the reaction was stopped with 50 μL of 25% acetic acid after incubation for 6 mins. Absorbance was measured at 405–410 nm on the Biorad microplate reader (Model no. 680). The endotoxin concentration in the study samples were quantified based on the standard curve and results expressed as EU/ml.

### Fecal elastase assay

The enzyme was assayed in fresh fecal samples using the solid phase double-sandwich ELISA based kit (Code no. G09038; from Bioserve Diagnostics, Rostock, Germany) as per manufacturer’s instructions^33^. The assay utilized two polyclonal antibodies that recognize organ-specific human pancreatic elastase peptide sequences. Briefly, fecal samples were treated with an extraction buffer, and supernatants of extracted fecal samples were diluted with washing solution. Standards and diluted fecal samples (50 μL) were incubated in the wells coated with the first antibody for 60 mins at room temperature, followed by incubation with biotinylated anti-elastase antibody for 30 mins and treatment with 50 μl of streptavidin peroxidase conjugate and 100 μL of substrate solution. The enzymatic reaction was stopped by adding 100 μL of stop solution and absorbance in each well was measured at 450 nm in a microplate reader (BioRad Model no. 680). The fecal elastase concentration was quantified based on the standard curve and expressed as μg/g stool.

### Isolation of fecal bacterial DNA

Fresh fecal specimens, collected in sterile containers, were immediately processed for DNA extraction. Genomic DNA was isolated from the stool specimen (200 mg) using QIAGEN (Germany) mini stool DNA isolation kit (Cat. No 81504) as per the manufacturer’s instruction. DNA concentration was determined using the Nano drop 2000 spectrophotometer (Thermo Scientific, IL, USA) and the purity was recorded based on absorbance at A_260_/A_280_. After quantity and quality check, the isolated DNA was stored at −80 °C till sequencing.

### Next generation sequencing on Illumina MiSeq platform

We subjected metagenomic DNA from the 40 samples to Next Generation Sequencing (NGS) at Xcelris Genomics (Ahmedabad, India). The V3-V4 region of 16S rDNA amplicon was sequenced on the Illumina MiSeq platform to analyse the bacterial diversity. The NexteraXT Index kit (Illumina Inc., USA) was used to generate a MiSeq amplicon library as per the 16S metagenomic sequencing library preparation protocol (Part # 15044223 Rev. B). Two primers (V3-forward: 5′CCTACGGGNGGCWGCAG3′ and V4-reverse: 5′GACTACHVGGGTATCTAATCC3′) were designed and synthesized for the amplification of V3–V4 region of 16S rDNA gene. Amplicons were then ligated with Illumina adaptors and i5 and i7 primers were used to amplify them. The amplicon libraries were purified using 1X Ampure XP beads and checked on Agilent DNA 1000 chip on bioanalyzer 2100 and further quantified on fluorometer by Qubit DNA HS Assay kit (Life Technologies, India). After obtaining the Qubit concentration for the library and the mean peak size (~600 to ~630 bp) from bioanalyser profile, 600 μl of 10 ρM pooled libraries (spiked with 5% 12.5 ρM PhiX Control) was loaded into MiSeq reagent cartridge for cluster generation involving hybridization of template molecules onto the oligonucleotide-coated surface of the flow cell. Immobilized template copies were amplified by bridge amplification to generate clonal clusters. Barcode and sequencing primers were trimmed from sequences. The trimmed sequences were saved as FASTQ files, which were then uploaded to Metagenomic RAST server (MG-RAST, Version 3.6)^34^. Low quality regions of FASTQ data were removed using Solexa QA. Sequences with an average Phred score lower than 25, containing ambiguous bases, homopolymer run of greater than 6, those having mismatches in primers, or sequence length shorter than 100 bp were also removed. Taxonomic assignment was carried out with 97% homology match in the MG-RAST server within Ribosomal Database Project (RDP). Bacterial abundance data at phylum, class, order, family, genus and species levels were downloaded from the server. Functional traits of each microbiota sample were assigned based on known genomes and prediction using the KEGG orthology (KO)^35^.

### Statistical analyses

Bacterial richness, alpha diversity and beta diversity was expressed as Chao 1, Shannon index and Whitaker index respectively^36^. Rarefaction curves were constructed for individual samples in the MG-RAST server. We performed the primary comparisons of intestinal microbial attributes between the controls, CP without and CP with diabetes. Principal component analysis (PCA) was performed using the Paleontological STatistics (PAST) software (Mac version 3.11) using the species level relative abundance data after normalization. Significance testing for the between-group clustering of the samples in PCA was carried out in PAST with one-way analysis of similarities (ANOSIM) using Monte Carlo simulation (10,000 replications). Furthermore, agglomerative hierarchical cluster analysis was performed with boot strapping to 1000 in order to ascertain distance based on Euclidean similarity matrix (by Ward’s method) between the individual samples. Abundance of different inter-group taxa and functional pathways, and correlations were represented as heat maps, and scatter plots that were constructed in the Plotly for R (version 2.0) platform and MS Excel for Mac 2011 (version 14.1.0). We expressed correlation between bacterial abundance and plasma glucose, endotoxin levels, and markers of exocrine insufficiency/malnutrition as Pearson and Spearman coefficient, as applicable. The Shapiro Wilk test was used to evaluate normalcy of data. Continuous data were presented as mean [standard deviation (SD)] and were analyzed using the non-parametric Kruskal-Wallis test or Mann Whitney U tests, as appropriate. Categorical data were presented as proportion. All statistical analyses were performed in the Statistical Package of Social Scientists (SPSS) (IBM SPSS 20, SPSS Inc, Chicago, IL). Bonferroni correction was applied for multiple testing and the adjusted ‘p’ value [denoted as p (corr.)] of <0.05 was considered statistically significant.

## Additional Information

**How to cite this article**: Manasa Jandhyala, S. *et al*. Altered intestinal microbiota in patients with chronic pancreatitis: implications in diabetes and metabolic abnormalities. *Sci. Rep.* **7**, 43640; doi: 10.1038/srep43640 (2017).

**Publisher's note:** Springer Nature remains neutral with regard to jurisdictional claims in published maps and institutional affiliations.

## Supplementary Material

Supplementary Information

## Figures and Tables

**Table 1:** Demographic, disease, nutritional and diabetic status of controls and patients with CP.

		Healthy controls (n = 10)	Chronic pancreatitis without diabetes (n = 16)	Chronic pancreatitis with diabetes (n = 14)	‘p’ value
Demographic characteristics	Age (yrs.) (Mean; SD)	42.3 (13.9)	30.8 (9.9)	34.8 (11.3)	0.05
	Male gender [n (%)]	7 (70.0)	11 (68.8)	11 (78.6)	0.82
	Non-vegetarian diet (n; %)	10 (100)	12 (75.0)	10 (71.4)	0.19
Disease (CP) characteristics	Duration of CP diagnosis at enrolment (yrs) (Mean; SD)	N/A	4.3 (1.6)	6.9 (2.4)	0.001
	Etiology (Alcohol/Idiopathic) (n; %)	N/A	4 (25.0)/12 (75.0)	3 (21.4)/11 (78.6)	0.99
	Gross pancreatic atrophy (n; %)	N/A	9 (56.3)	9 (64.3)	0.29
	Pancreatic calcification/calculi (n; %)	N/A	10 (62.5)	8 (57.1)	0.76
	Pancreatic pseudocyst (n; %)	N/A	1 (6.3)	0 (0)	0.53
	Biliary stricture (n; %)	N/A	2 (12.5)	1 (7.1)	0.55
	Endoscopic/Surgical intervention (n; %)	N/A	7 (43.8)	11 (78.6)	0.07
	Pancreatic exocrine insufficiency [n (%)]	N/A	11 (68.8)	13 (92.8)	0.17
Nutritional characteristics	Subjective global assessment (A/B/C) [n; %]	10 (100)/0 (0)/0 (0)	10 (62.5)/5 (31.3)/1 (6.3)	3 (21.4)/7 (50)/ 4 (28.6)	0.003
	BMI at presentation (Mean; SD)	21.5 (9.2)	19.7 (5.9)	18.7 (9.3)	0.74
	Haemoglobin (gm/dl) (Mean; SD)	13.7 (1.8)	13.7 (1.7)	12.3 (2.0)	0.08
	Serum calcium (mg/dl) (Mean; SD)	9.6 (0.4)	9.4 (0.9)	9.6 (0.3)	0.62
	Pre-albumin (mg/dL (Mean; SD)	31.56 (8.6)	27.88 (6.0)	18.1 (5.7)	<0.0001
	Transferrin (mg/dL) (Mean; SD)	288.9 (33.6)	302.11 (62.7)	192.07 (53.8)	<0.0001
	Vitamin D (U/L) (Mean; SD)	41.0 (14.0)	15.4 (8.5)	12.8 (4.7)	<0.0001
	Vitamin B12 (pg/mL) (Mean; SD)	328.4 (107.6)	352.31 (219.1)	243.7 (119.6)	0.19
Diabetic status	Duration of diabetes (yrs) (Mean; SD)	N/A	N/A	1.5 (1.3)	—
	On insulin at the time of enrolment (n; %)	N/A	N/A	4 (28.6)	—
	Fasting blood glucose (mg/dL) (Mean; SD)	90.5 (8.9)	84.3 (10.9)	209.5 (48.3)	<0.0001
	Post-prandial blood glucose (mg/dL) (Mean; SD)	101.8 (6.7)	103.8 (9.1)	291.6 (62.6)	<0.0001
	Glycosylated haemoglobin (Mean %; SD)	5.4 (0.9)	5.3 (0.8)	8.1 (0.7)	<0.0001
