# Supplementary material for: Altered intestinal microbiota in patients with chronic pancreatitis: implications in diabetes and metabolic abnormalities
Source: Sci Rep. 2017 Mar 3;7:43640. doi: 10.1038/srep43640 (PMC5334648; doi:10.1038/srep43640)
Supplement: Supplementary Information [file srep43640-s1.pdf]

Supplementary materials:

Altered intestinal microbiota in patients with chronic pancreatitis: implications in diabetes and metabolic abnormalities.

Sai Manasa Jandhayala, A. Madhulika, G. Deepika, G Venkat Rao, D Nageshwar Reddy, Chivukula Subramanyam, Mitnala Sasikala, Rupjyoti Talukdar.

Supplementary Table S1: Dietary details of individual controls and patients.

The proximate principles were calculated using nutritive value of Indian Foods. The numbers from 1-7 in the columns showing individual dietary components indicate the number of times the patient's/control's included those food items in a week; 8 indicates use once a fortnight; 9 indicates once a month; and 10 indicates never included. Information on dietary intake was obtained from 24hr recall and food frequency questionnaire.

Group	ID	Energy (kcal)	Protein (gm)	Carbohy drate (gm)	Fat (gm)	Rice	Wheat	Other cereals	Dals	Lentils	Others pulses	Leafy vegetabl es	Roots and tubers	Other vegetabl es	Fruits	Nuts and oilseeds	Milk and milk products	Fats and oils	Meat product
Control1	4600001.3	1364	55	177	41	7	3	10	6	1	1	2	2	2	7	1	7	7	2
Control2	4584129.3	1373	47	181	42	7	2	1	7	10	10	3	6	6	2	9	7	7	7
Control3	4600002.3	1396	44	195	35	6	1	1	5	1	10	2	6	3	2	6	6	7	7
Control4	4622474.3	1238	55	124	29	7	1	10	6	1	1	2	7	4	1	2	7	7	5
Control5	4584133.3	1271	41	182	35	7	2	10	7	1	10	2	3	6	1	5	7	7	4
Control6	4600020.3	1375	50	111	21	6	2	10	6	1	1	2	2	4	3	6	7	7	5
Control7	4584456.3	1151	42	153	23	5	4	10	5	10	1	2	1	3	1	9	7	7	2
Control8	4584455.3	1248	45	195	21	7	6	10	6	10	1	3	1	1	2	9	7	7	2
Control9	4584593.3	1250	60	195	40	7	7	10	7	1	10	3	6	7	3	9	7	7	1
Control10	4600000.3	1170	50	240	38	7	1	10	7	1	2	2	6	3	6	9	7	7	2
CPND1	4622479.3	1107	50	233	21	6	6	10	2	1	10	1	2	1	2	8	7	7	10
CPND2	4584718.3	1127	34	171	33	7	7	10	1	1	10	2	3	8	8	10	10	7	10
CPND3	4600013.3	1042	52	208	23	7	2	10	7	10	10	1	1	1	3	7	1	7	2
CPND4	4584253.3	1089	43	216	21	7	7	10	7	10	1	10	1	7	1	1	7	7	7
CPND5	4584721.3	1844	50	237	30	7	7	10	7	7	7	7	7	1	1	10	7	7	1
CPND6	4584262.3	1411	46	153	20	7	10	10	7	10	10	8	1	8	9	9	3	7	4
CPND7	4600014.3	1080	34	316	23	7	7	10	2	3	10	10	7	6	1	9	7	7	2
CPND8	4622481.3	1196	67	229	46	6	5	10	1	10	1	2	6	5	7	1	1	7	8
CPND9	4600015.3	1156	41	179	22	7	6	10	6	1	1	2	4	6	6	2	1	7	7
CPND10	4622480.3	1055	28	150	25	7	10	10	2	7	10	10	7	7	7	10	10	7	10
CPND11	4584237.3	1799	55	297	40	7	1	10	8	3	10	1	4	1	3	8	1	7	1
CPND12	4584614.3	1245	55	134	16	7	9	10	1	1	10	1	2	4	4	9	2	7	10
CPND13	4600011.3	1216	30	183	32	6	5	10	3	2	6	3	3	3	5	3	3	7	7
CPND14	4600012.3	918	13	103	10	5	5	10	3	3	6	3	2	3	6	2	2	7	5
CPND15	4600016.3	1380	62	191	38	7	6	10	3	2	6	3	2	3	4	1	4	7	4
CPND16	4584261.3	1142	32	258	33	7	2	10	7	10	10	9	8	1	2	1	1	7	7
CPD1	4600008.3	883	35	166	24	7	7	9	7	9	10	1	2	2	9	6	8	7	2
CPD2	4600009.3	1799	74	218	45	7	8	10	7	10	10	9	8	2	1	10	10	7	10
CPD3	4622475.3	1020	36	275	27	7	7	10	7	10	10	8	1	7	1	1	1	7	2
CPD4	4600010.3	1195	46	230	25	7	10	7	7	7	10	9	4	4	4	1	5	7	4
CPD5	4584457.3	1040	30	194	19	7	6	10	6	3	1	9	6	6	6	9	6	7	10
CPD6	4584598.3	1682	68	255	47	6	8	10	5	3	1	9	6	6	4	8	6	7	10
CPD7	4622476.3	1401	44	207	30	7	7	10	6	3	1	9	6	2	3	9	4	7	3
CPD8	4622477.3	1038	40	182	21	7	7	9	5	2	1	9	1	2	3	8	3	7	8
CPD9	4622478.3	1134	37	186	22	5	5	9	7	2	1	9	2	2	5	4	2	7	10
CPD10	4600003.3	1630	51	275	27	6	6	9	7	2	10	3	2	10	4	10	5	7	6
CPD11	4600004.3	1353	27	325	29	7	4	10	7	1	1	3	3	2	2	12	7	7	5
CPD12	4600005.3	1654	49	430	37	7	6	10	7	1	10	3	1	1	1	6	6	7	5
CPD 13	4600006.3	1164	39	180	34	7	5	9	7	1	10	3	1	2	9	5	1	7	3
CPD 14	4600007.3	1252	46	218	18	7	7	9	7	2	10	3	3	3	8	4	1	7	2

Supplementary Table S2: Sequence characteristics, metagenomic quantity/quality, richness and diversity of intestinal microbiota.

	Patient ID	Disease category	Post QC sequence count	Post QC sequence length (bp ± SD)	Metagenomic quantity (ng/µl)	Metagenomic quality (A260/280)	Chao1	Shannon index
Control1	4600001.3	Healthy control (n=10)	346,009	422 ± 73	242.4	1.82	111	2.25
Control2	4584129.3		515,182	364 ± 147	214.3	2.15	82	2.47
Control3	4600002.3		330,699	371 ± 119	647.8	2.13	102	2.00
Control4	4622474.3		891,169	388 ± 145	331.3	2.14	101	2.04
Control5	4584133.3		387,765	356 ± 152	196.2	2.12	116	2.08
Control6	4600020.3		337,751	343 ± 130	381.2	2.13	94	2.42
Control7	4584456.3		625,510	419 ± 113	317.9	2.17	121	2.28
Control8	4584455.3		450,811	414 ± 112	245.5	2.15	93	2.15
Control9	4584593.3		423,013	420 ± 113	285.3	2.07	102	1.98
Control10	4600000.3		322,656	420 ± 75	181.5	2.17	113	1.84
CPND1	4622479.3	Chronic pancreatitis without diabetes (n=16)	571,360	410 ± 116	222.5	2.13	134	2.02
CPND2	4584718.3		411,513	413 ± 117	361.9	2.12	51	2.64
CPND3	4600013.3		279,982	451 ± 15	178.6	2.13	41	2.39
CPND4	4584253.3		775,700	370 ± 148	407.8	2.13	50	2.15
CPND5	4584721.3		415,009	413 ± 111	180.9	2.19	63	2.04
CPND6	4584262.3		610,189	370 ± 144	561.5	2.12	69	2.62
CPND7	4600014.3		55,180	428 ± 144	290.5	2.13	66	1.52
CPND8	4622481.3		604,179	428 ± 99	378.9	2.17	117	2.23
CPND9	4600015.3		340,323	442 ± 11	157.2	2.12	107	2.12
CPND10	4622480.3		973,267	410 ± 125	110.3	2.17	90	2.35
CPND11	4584237.3		489,279	364 ± 150	228.8	2.13	107	2.33
CPND12	4584614.3		557,622	411 ± 114	233.1	2.19	118	2.24
CPND13	4600011.3		276,662	423 ± 72	246.1	2.16	91	2.06
CPND14	4600012.3		175,232	420 ± 66	289.3	2.14	104	2.38
CPND15	4600016.3		246,822	380 ± 122	256.3	1.71	94	1.78
CPND16	4584261.3		11,575	198 ± 131	171.4	2.15	95	1.99
CPD1	4600008.3	Chronic pancreatitis with diabetes (n=14)	17,618	360 ± 85	247.6	1.88	116	2.19
CPD2	4600009.3		351,046	371 ± 120	112.7	2.07	39	2.18
CPD3	4622475.3		547,780	402 ± 122	310.2	1.86	74	2.05
CPD4	4600010.3		319,333	405 ± 82	213.1	2.22	29	1.82
CPD5	4584457.3		483,061	414 ± 116	486.6	2.02	69	2.14
CPD6	4584598.3		445,616	419 ± 114	644.5	2.16	58	1.57
CPD7	4622476.3		671,154	417 ± 114	365.3	2.12	114	2.18
CPD8	4622477.3		401,007	410 ± 111	280.4	2.16	42	1.66
CPD9	4622478.3		848,237	419 ± 115	304.9	2.04	63	1.96
CPD10	4600003.3		227,616	437 ± 53	370.3	2.11	49	1.87
CPD11	4600004.3		265,847	440 ± 46	264.4	2.16	46	1.79
CPD12	4600005.3		312,204	441 ± 48	239.1	2.15	41	2.15
CPD13	4600006.3		232,960	440 ± 58	474.2	2.11	92	2.06
CPD14	4600007.3		187,466	444 ± 47	274.2	2.03	44	2.08

Supplementary Table S3: Comparison of study parameters between individual groups.

		Healthy control v/s CP without diabetes	Healthy control v/s CP with diabetes	CP without diabetes v/s CP with diabetes
**Chao 1**	Median (IQR)	101.5 (22) v/s 92.5 (43)	101.5 (22) v/s 53.5.5 (37)	92.5 (43) v/s 53.5.5 (37)
	p value	0.17	**0.002***	**0.02***
**Shannon index**	Median (IQR)	2.2 (0.33) v/s 2.2 (0.35)	2.2 (0.33) v/s 2.05 (0.34)	2.2 (0.35) v/s 2.05 (0.34)
	p value	0.78	**0.04***	**0.05***
**Firmicutes: Bacteroidetes**	Median (IQR)	1.01 (0.91) v/s 1.5 (0.95)	1.01 (0.91) v/s 1.89 (2.12)	1.5 (0.95) v/s 1.89 (2.12)
	p value	0.17	**0.04***	0.37
***Fecalibacterium praunitszii* relative abundance**	Median (IQR)	0.17 (0.12) v/s 0.12 (0.03)	0.17 (0.12) v/s 0.09 (0.03)	0.12 (0.03) v/s 0.09 (0.03)
	p value	**0.03***	**0.001***	**0.005***
***Ruminococcus bromii* relative abundance**	Median (IQR)	0.09 (0.04) v/s 0.02 (0.06)	0.09 (0.04) v/s 0.004 (0.02)	0.02 (0.06) v/s 0.004 (0.02)
	p value	**0.01***	**0.001***	**0.04***
**LPS synthetic pathway abundance**	Median (IQR)	0.0001 (0.004) v/s 0.09 (0.08)	0.0001 (0.004) v/s 0.22 (0.16)	0.09 (0.08) v/s 0.22 (0.16)
	p value	**0.006***	**0.01***	0.06
**Plasma endotoxin level (EU/mL)**	Median (IQR)	0.32 (0.09) v/s 0.51 (0.11)	0.32 (0.09) v/s 1.01 (0.36)	0.51 (0.11) v/s 1.01 (0.36)
	p value	**0.0007***	**0.0002***	**0.001***

Mann Whitney U test was used for the comparisons; * indicates statistical significance

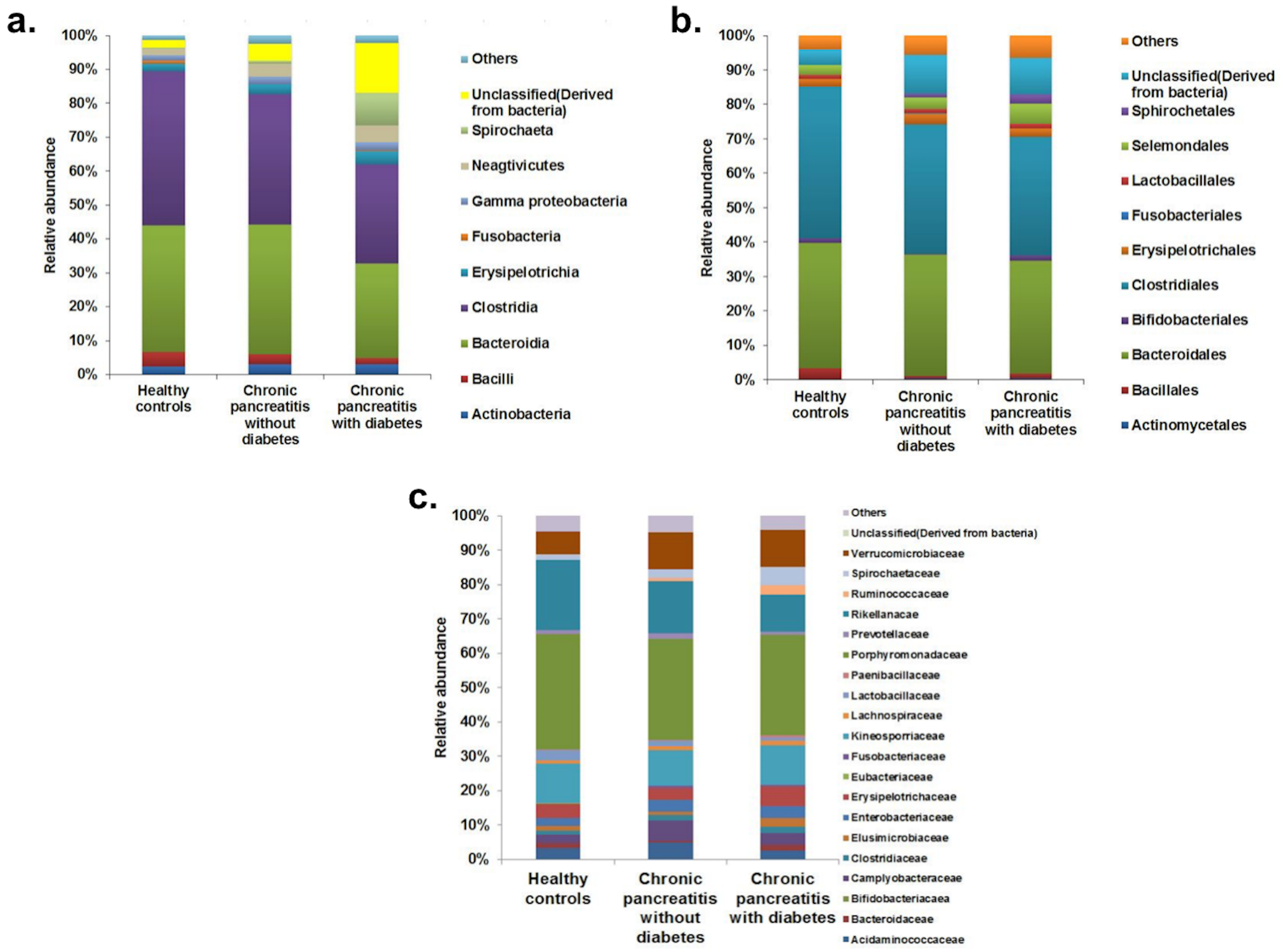

Supplementary figure S1: Stacked bar diagrams depicting the differences in relative abundances of gut microbiota between healthy controls, CP without diabetes and CP with diabetes at the **(a)** class, **(b)** family and **(c)** order level taxa. There were no statistically significant differences between any of the organisms between the three groups.

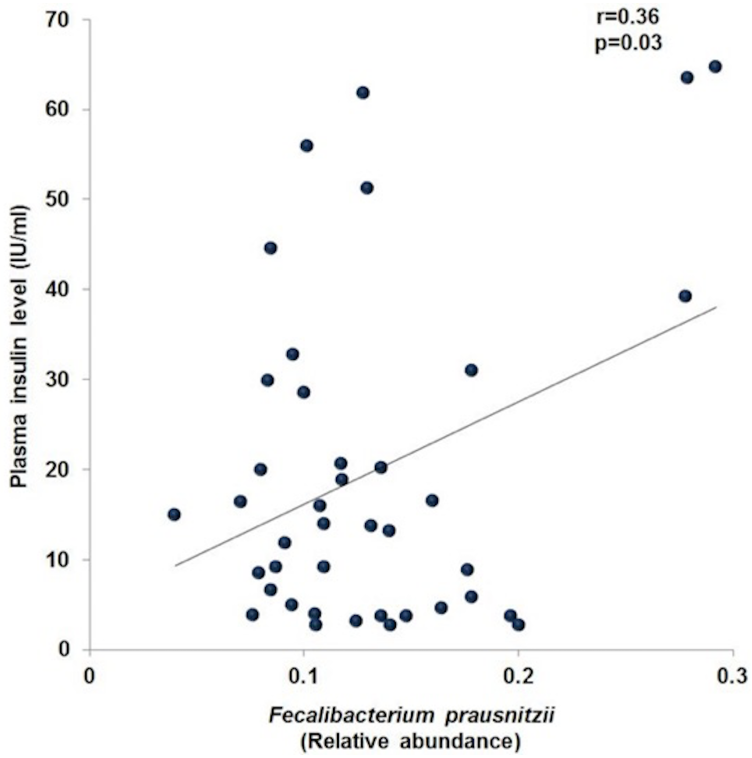

Supplementary figure S2: Significant positive correlation of *Faecalibacterium prausnitzii* abundance with plasma insulin levels.

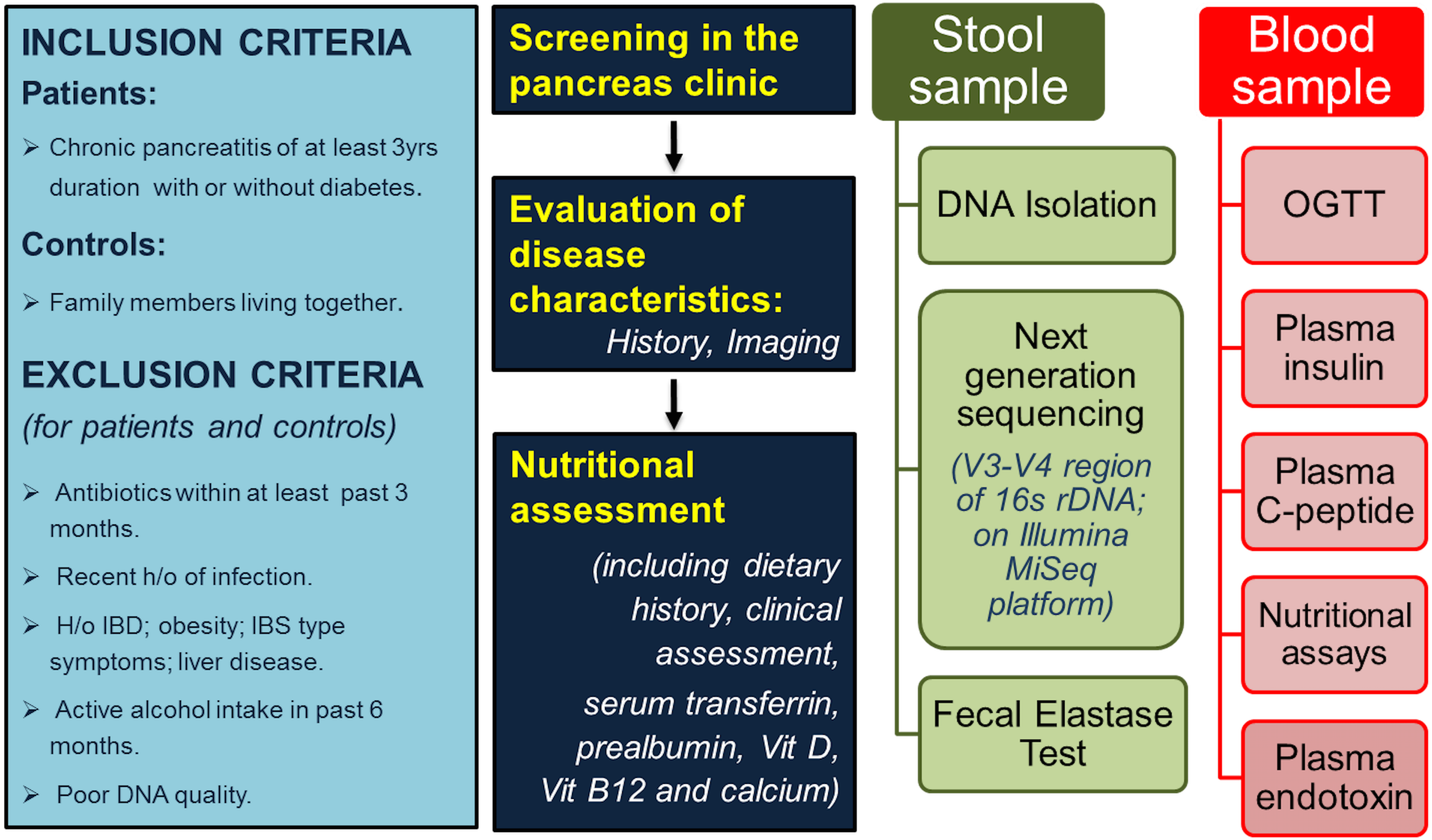

Supplementary figure S3: Schematic representation of study design.
